# 500 Anticoagulant Use Is Associated with Different Outcomes in Pediatric and Adult Burn Patients

**DOI:** 10.1093/jbcr/irae036.135

**Published:** 2024-04-17

**Authors:** Julia Kleinhapl, Kristine Knappskog, Amina E I ayadi, Juquan Song, oscar E Suman-Vejas, Ludwik K Branski, Steven E Wolf

**Affiliations:** Department of Surgery, Division of Surgical Sciences, Medical Branch, University of Texas, Galveston, TX, USA; Division of Plastic, Aesthetic and Reconstructive Surgery, Department of Surgery, Medical University of Graz, Graz, Austria, Galveston, TX; University of Texas Medical Branch at Galveston and University of Bergen, Galveston, TX; University of Texas Medical Branch, Galveston, TX; University of Texas Medical Branch at Galveston, Galveston, TX; University of Texas Medical Branch, Blocker Burn Unit, Galveston, TX; Department of Surgery, Division of Surgical Sciences, Medical Branch, University of Texas, Galveston, TX, USA; Division of Plastic, Aesthetic and Reconstructive Surgery, Department of Surgery, Medical University of Graz, Graz, Austria, Galveston, TX; University of Texas Medical Branch at Galveston and University of Bergen, Galveston, TX; University of Texas Medical Branch, Galveston, TX; University of Texas Medical Branch at Galveston, Galveston, TX; University of Texas Medical Branch, Blocker Burn Unit, Galveston, TX; Department of Surgery, Division of Surgical Sciences, Medical Branch, University of Texas, Galveston, TX, USA; Division of Plastic, Aesthetic and Reconstructive Surgery, Department of Surgery, Medical University of Graz, Graz, Austria, Galveston, TX; University of Texas Medical Branch at Galveston and University of Bergen, Galveston, TX; University of Texas Medical Branch, Galveston, TX; University of Texas Medical Branch at Galveston, Galveston, TX; University of Texas Medical Branch, Blocker Burn Unit, Galveston, TX; Department of Surgery, Division of Surgical Sciences, Medical Branch, University of Texas, Galveston, TX, USA; Division of Plastic, Aesthetic and Reconstructive Surgery, Department of Surgery, Medical University of Graz, Graz, Austria, Galveston, TX; University of Texas Medical Branch at Galveston and University of Bergen, Galveston, TX; University of Texas Medical Branch, Galveston, TX; University of Texas Medical Branch at Galveston, Galveston, TX; University of Texas Medical Branch, Blocker Burn Unit, Galveston, TX; Department of Surgery, Division of Surgical Sciences, Medical Branch, University of Texas, Galveston, TX, USA; Division of Plastic, Aesthetic and Reconstructive Surgery, Department of Surgery, Medical University of Graz, Graz, Austria, Galveston, TX; University of Texas Medical Branch at Galveston and University of Bergen, Galveston, TX; University of Texas Medical Branch, Galveston, TX; University of Texas Medical Branch at Galveston, Galveston, TX; University of Texas Medical Branch, Blocker Burn Unit, Galveston, TX; Department of Surgery, Division of Surgical Sciences, Medical Branch, University of Texas, Galveston, TX, USA; Division of Plastic, Aesthetic and Reconstructive Surgery, Department of Surgery, Medical University of Graz, Graz, Austria, Galveston, TX; University of Texas Medical Branch at Galveston and University of Bergen, Galveston, TX; University of Texas Medical Branch, Galveston, TX; University of Texas Medical Branch at Galveston, Galveston, TX; University of Texas Medical Branch, Blocker Burn Unit, Galveston, TX; Department of Surgery, Division of Surgical Sciences, Medical Branch, University of Texas, Galveston, TX, USA; Division of Plastic, Aesthetic and Reconstructive Surgery, Department of Surgery, Medical University of Graz, Graz, Austria, Galveston, TX; University of Texas Medical Branch at Galveston and University of Bergen, Galveston, TX; University of Texas Medical Branch, Galveston, TX; University of Texas Medical Branch at Galveston, Galveston, TX; University of Texas Medical Branch, Blocker Burn Unit, Galveston, TX

## Abstract

**Introduction:**

Anticoagulation therapy in pediatric burn patients remains an under-researched area of burn medicine lacking guidelines or scores to establish standardized therapy. The aim of this study is to evaluate the use of anticoagulants in pediatric compared to adult burn patients and to identify the most frequently used drug classes.

**Methods:**

A large database was searched to create two cohorts of burn patients with more than 10% TBSA. Pediatric patients (age 0-17) were compared to adult burn patients (18-100) after propensity score matching was carried out for gender, ethnicity, and race.

Venous thromboembolic events (VTE), mortality, and bleeding events were defined as outcome variables. Data analysis was performed with the tools of descriptive statistics. Risk and odds ratios were presented with significance set at p< 0.05. We also examined and compared the number of cases in which burn victims were treated with anticoagulants and the frequency distribution of different anticoagulant classes. Anticoagulants were divided into 4 groups: heparins, factor Xa-inhibitors, vitamin K antagonists, and thrombin inhibitors.

**Results:**

After propensity score matching, each of the cohorts included 5630 patients. 5.5% and therefore significantly (p< 0.001) more adult patients showed thromboembolic complications compared to 1.4% of pediatric patients, with RR 0.26 (95% CI 0.20-0.33) and OR 0.24 (95% CI 0.19-0.31). Bleeding events occurred in 4.9% of adult patients, which is more than twice as common as in pediatric patients (2.1%), with RR 0.42 (95% CI 0.34-0.52) and OR 0.41 (95% CI 0.33-0.51). With 5.8% vs. 1.5% deaths in adults compared to pediatrics, mortality was also significantly higher (p< 0.001) in the adult cohort, showing RR of 0.27 (95% CI 0.21-0.34) and OR of 0.26 (95% CI 0.20-0.32).

While 33.4% of adult burn patients were treated with anticoagulants within the first week after burn, only 6.7% of the pediatric burn population were treated. Further, the most frequently used drug group in both adult and pediatric burn patients was heparins, given in over 90% of the cases. Other drug classes were used in less than 10% of cases.

**Conclusions:**

Anticoagulant treatment within the first 7 days after burn is more likely administered to adults than to pediatric patients. However, in most cases, heparins were the drug of choice in both groups. Bleeding events, the occurrence of VTE, and mortality was significantly higher in adult burn patients.

**Applicability of Research to Practice:**

The discrepancy in the use of anticoagulants between adults and pediatrics in burn care highlights the need for larger studies to develop standardized guidelines for the use of different anticoagulants in pediatric burn patients.

